# Knowledge of and perception towards eclampsia among women and men in Unguja Island, Zanzibar: A qualitative study

**DOI:** 10.1371/journal.pone.0313536

**Published:** 2025-01-15

**Authors:** Nassra Is-hak Yussuf, Jelle Stekelenburg, Michael Johnson Mahande, Rachel Nathaniel Manongi

**Affiliations:** 1 Kilimanjaro Christian Medical University College, Moshi, Tanzania; 2 Department of Nursing and Midwifery, School of Health and Medical Sciences, State University of Zanzibar, Tanzania; 3 Department of Health Sciences, Global Health, University Medical Centre Groningen, University of Groningen, Groningen, the Netherlands; 4 Department Obstetrics & Gynaecology, Leeuwarden Medical Centre, Leeuwarden, the Netherlands; 5 Department of Epidemiology and Biostatistics, Kilimanjaro Christian Medical University College, Moshi, Tanzania; 6 Institute of Public Health, Kilimanjaro Christian Medical University College, Moshi, Tanzania; National Research Centre, EGYPT

## Abstract

**Background:**

Eclampsia is among the primary causes of maternal and perinatal morbidity and mortality in Zanzibar. Many women and men are not aware of the signs, symptoms and causes of eclampsia and may have different explanatory models. Therefore, this study aimed to describe the community understanding of pre-eclampsia, as a key stage to improve maternal and perinatal health in Unguja Island, Zanzibar.

**Methods:**

A qualitative study design of six focus group discussions (FDGs) was performed using a focus group interview guide; 51 male and female **r**espondents at three selected wards of Unguja Island, Zanzibar participated. Thematic analysis was applied using Qualitative data analysis (QDA Miner Lite software version 2.0.9).

**Results:**

Overall participants demonstrated a lack of understanding regarding eclampsia. The majority of participants perceived that there are certain things related to eclampsia: Mjusi or Mdudu (devil/satanic), superstitious issues, tension/stress from partners, food consumption, eating slaughtered meat like chicken, cow and goat during pregnancy, nice smelling lotion, soap and perfumes have been stated to be the causes of eclampsia. Spiritual and traditional remedies were mentioned as a treatment for eclampsia, including herbs, makombe (written verses of the Qur’an) and steaming (smoked dry leaves). Local names of eclampsia used by the communities, in urban and rural areas are mjusi (lizard) or mdudu (bug), which means the devil enters a woman’s body, especially during pregnancy or within 42 days post-delivery.

**Conclusion:**

Most participants had a lack of understanding of eclampsia and perceived that there are traditional and spiritual issues related to its causes, attributed risks and clinical presentation, which makes them rely more on traditional and spiritual treatments. Therefore, health educational programs in the community setting and at the antenatal clinics, aiming at improving knowledge and dismissing myths and misperceptions regarding eclampsia, are recommended in rural and urban areas of Unguja Zanzibar.

## Introduction

Pregnancy-induced hypertension, including (pre-)eclampsia, is a major health problem and the main cause of maternal and perinatal morbidity and mortality worldwide [1, 2]. It is a serious disorder related to pregnancy and increases the risk of cardiovascular disease in later life [1, 3].

Globally about 76,000 women and 500,000 babies die each year from childbirth complications including eclampsia. Eclampsia continues to be the leading cause of maternal and perinatal mortality and morbidity throughout the world [4]. In low-income countries, particularly in sub-Saharan Africa, maternal mortality remains a public health concern: about 99% of the estimated maternal deaths worldwide are in developing countries, and most could have been prevented [5]. Eclampsia contributes about 2% to 15% of direct maternal deaths and nearly a quarter of stillbirths and newborn deaths in developing countries [6].

Eclampsia is defined as seizures that cannot be attributable to other causes in a woman with preeclampsia [7], characterized by the new onset of hypertension, proteinuria and seizures, or any other organ manifestation, after 20 weeks of gestation in a previous normotensive woman [8].

A study in Pakistan revealed that most of the participants perceived eclampsia as attributed to excessive thinking/stress, lack of rest and domestic problems [9]. Likewise, a study done in India found that anaemia, poor medical adherence, lack of tetanus toxoid immunization and exposure in pregnancy to fire or water were thought to be among the causes of eclampsia [10]. Studies done in Ethiopia, southern Mozambique and Nigeria revealed that eclampsia was not known in the communities. or eclampsia was perceived as a stress-induced condition, or attributed to prolonged exposure to cold [11–13].

Tanzania, like other developing countries, continues to have a high maternal mortality ratio accounting for 500 deaths per 100,000 live births, with a fertility rate of 4.8 children per woman and a neonatal mortality rate of 24 per 1000 live births [14]. Preeclampsia accounted for 20% of maternal mortality and was found to be the main cause of maternal death [15–17]. In Dodoma, Tanzania, a study found that nearly half of respondents were not aware of eclampsia and its associated risk factors [18]. In Tanzania, it was found that the knowledge gap on eclampsia was among the contributing factors to the high maternal and perinatal morbidity and mortality. The Millennium Development Goal (MDG), target was to reduce the maternal mortality ratio to 133 per 100,000 live births by 2015. Now, both global and national efforts are being made to reduce this ratio to fewer than 70 maternal deaths [15].

The prevalence of severe preeclampsia among postpartum women in Zanzibar is 26.3%, and more than half of the respondents did not know about eclampsia and its complications [18, 19].

Zanzibar has made efforts to increase the number of health facilities and to ensure the number of skilled personnel is sufficient and competent to prevent maternal and perinatal morbidity and mortality. However, the nature of eclampsia remains unknown by the communities which causes increases in maternal morbidity and mortality in Tanzania [19]. Therefore, the goal of reducing maternal and perinatal mortality caused by eclampsia might not be achieved if the community members are not aware of eclampsia and its effects. Thus, this study aimed to explore the knowledge and perception towards eclampsia among women aged 18–49 years and men in Unguja Island, Zanzibar.

## Methods

### Study design, study area and study period

A phenomenological study approach was applied to explore knowledge and perception towards eclampsia among women aged 18–49 years and men in Unguja Island, Zanzibar. The study was conducted in three specific study areas: Kwabitihamrani, Pitanazako, and Tasani, which are situated in three different regions of Unguja Island. These regions are the Urban region of Unguja, the North "A" region of Unguja, and the South region of Unguja in Zanzibar.

### Study population and sampling technique

The study included 51 women of childbearing age and adult men (>18), married or unmarried, who were not couples or married to each other and eligible to participate in the study from three selected wards of Unguja Island, Zanzibar. A multi-stage sampling strategy was employed purposively to identify regions and districts, and simple random sampling was done to select villages within the wards (a subdivision of a large administrative area within the district). A convenience sampling was employed to recruit participants for the Focus Group Discussions (FGD), in collaboration with village/community leaders who had connected with the community health management team. Community members were identified and invited to participate based on criteria set by the researchers. FGDs were held separately for women and men at school venues. Six FGDs were conducted with eight to ten participants until saturation was reached.

### Data collection tool and data collection process

A semi-structured focus interview guide with ten questions was used to collect the information from the participants. Questions one to seven assessed knowledge and perception concerning eclampsia, questions eight to eleven assessed management and prevention of eclampsia, and questions twelve discussed the recommendations for eclampsia. The interview guide included a section on demographic characteristics of the participants: age, sex, marital status, occupation and level of education. The guide was prepared in English following the review of the literature [20]. Then the guide was translated to the Kiswahili language which is spoken as a primary language in the study area to ease the communication process. Open-ended questions were used to obtain information including Complications of eclampsia in pregnant women, the local name used as an alternative to eclampsia, their thoughts on the causes and risk factors and how the condition should be managed and prevented.

Before opening the discussion, all study participants were informed about the aim of the study. The discussions were conducted in the local language, Kiswahili. In each FGD, study participants were seated in a semicircle sitting together with the PI and note taker. Before the discussion, all study participants were given an identification number to be used throughout the discussion. All FGDs were recorded using digital voice recorders. Written consent for recording their responses was requested from all the participants. Confidentiality was assured by deleting names as identification in the collected information. Each FGD lasted for about 50 to 60 minutes, the principal investigator (PI) moderated the discussion and a female nurse with experience in qualitative research took notes.

### Trustworthiness

To ensure the credibility and dependability of the results, after a review of relevant literature, the questionnaire tool was developed by the PI and it was then closely reviewed by supervisors to ensure that it covered the objectives of the study. The tool was translated to the local language. A pilot testing was performed at Mwera in the central district of Unguja Zanzibar two weeks before the actual data collection period. Nine questions were tested from the focus interview guide by performing two FGDs (with men and women). A detailed description, including the purpose of the study, was provided to participants, allowing them to share experiences, practices and beliefs concerning the topic. The transcription and translation were done soon after the data collection. Subsequently, the research findings were shared with the participants of the study to allow them to offer feedback on the interpretations that the researcher had made. The PI and the supervisor rechecked and modified the tool afterwards before the real data collection started. To ensure the consistency and completeness of the study, the PI checked field notes and audio recordings each day after the data collection for their clarity and saved the notes to the computer for analysis. The focus group interview guide (Knowledge of and perception towards eclampsia) and The COREQ (Consolidated criteria for Reporting Qualitative research) checklist are found in S1 File.

### Data analysis

**Fig 1:**
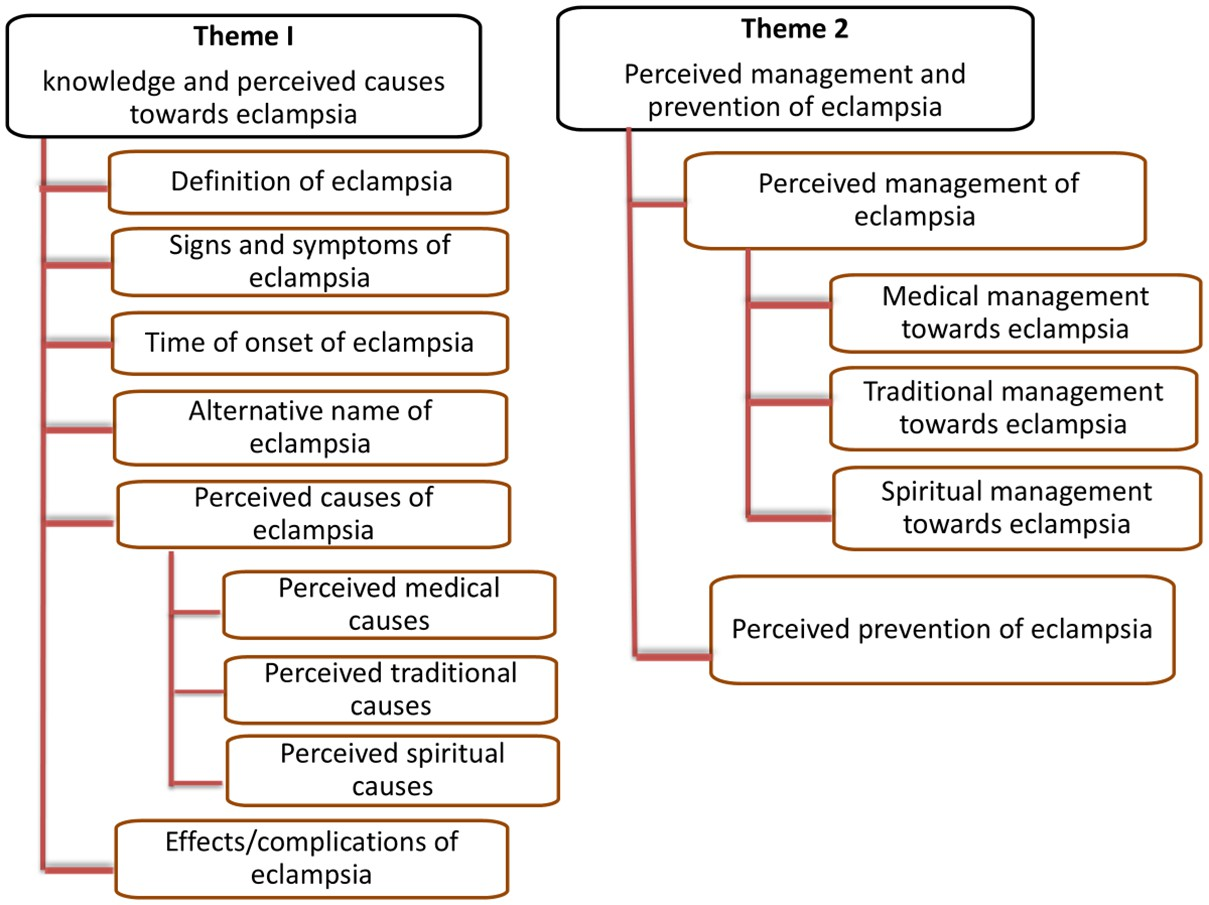
Thematic analysis categories.

A thematic approach was used to analyse the data. The audio records were transcribed into Kiswahili text and then translated into English by a language professional from the State University of Zanzibar. The first author (NY) and the research team crosschecked the transcribed notes. The first author (NY) did code by using QDA Miner Lite software to extract relevant information. Two independent researchers reviewed the codes independently (RM and VK). Using deductive reasoning, results were grouped into arranged categories of key themes (main and sub-themes) related to community knowledge of and perceptions towards eclampsia (Fig 1). The data were organized by coding into a meaningful element using the (QDA Miner Lite software) version 2.0.9. Saturation of the data was achieved when no more codes emerged from the data. The PI always checked for accuracy during the coding process to ensure that the meaning of units, codes and categories matched with emerging patterns. The discrepancies in the data were determined by the research team and agreed upon through discussion. After completion of the coding process, major issues were highlighted, appropriate themes were searched for categorization and the emerging themes were defined.

### Ethics approval and consent to participate

The study has been approved by the Kilimanjaro Christian Medical University College Research and Ethics Review Committee (certificate number 2495). Approval to conduct the study was also granted by the Zanzibar Medical Research Council (ZAHREC). Permission to involve the community was sought from the Second Vice President’s office of Zanzibar. A written consent form was obtained from participants to confirm their willingness to participate in the study. In terms of confidentiality, the consent forms, notebooks and audio-recorded tapes were kept confidential, and only study personnel had access to the materials. 

## Results

### Characteristics of the study participants

Six FGDs were conducted; three with men and three with women aged 24 to 49 years. The mean age of the male participants was 43.3 years (range: 26–71 years), while the mean age of female participants was 36.4 years (range: 24–49). More than half (67%) of the study participants were aged 24–49 years. Most of the study participants (92%) were married, the majority (88%) had completed secondary education, and 12% had primary education. More than half (55%), of the study participants, were self–employed and 10%, were employed by the government (Table 1).

**Table 1:** Socio-demographic characteristics of respondents (n = 51).

Variable	Frequency (n)	Percentage (%)
Gender		
Male	26	51%
Female	25	49%
Age group (years)		
Male 26–71 years	26	51%
Female 24–49 years	25	49%
Mean age (years) male: 43.3		
Mean age (years) female: 36.4		
Marital status		
Married	47	92%
Divorced	4	8%

### Themes of the study

The study has two main themes and sub-themes: 1). knowledge and perceived causes towards eclampsia covered the definition and signs and symptoms of eclampsia, onset and its alternative name of eclampsia, effects/complications and perceived causes of eclampsia. 2). Perceived management and prevention of eclampsia, covered the management and prevention of eclampsia.

The Fig 1. Describe the two main themes towards eclampsia which are knowledge and perceived causes and perceived management with prevention of eclampsia among community members.

#### Theme 1: Knowledge and perceived causes towards eclampsia

*Knowledge of eclampsia*. The study found that there is limited awareness of eclampsia and contributing factors among male and female participants. Subthemes that emerged under this theme were the definition of eclampsia and its occurrence, signs and symptoms, causes and effects.

***i) Meaning/ local name of eclampsia*.** The majority of participants, both males and females, responded on what they knew about eclampsia. According to them, eclampsia occurs during pregnancy and can happen during and/or after delivery, as narrated below by some of the participants.

*"On my side*, *[eclampsia] is when the body gets weak at the time of giving birth–is why she has seizures during childbirth*, *and on the day*, *she wants to give birth again*, *she experiences a little blood loss"*

*(R6-49 F*, *FGD 3)*

"*What I know is eclampsia is for a pregnant mother before or after delivery*"

*(R3-38 F*, *FGD 5)*

"*We understand it as a disease that a pregnant woman gets mostly during delivery*"

*(R1-30 M*, *FGD 6)*

*"When we say eclampsia*, *these are diseases that attack pregnant women; most of the time the eyes are swelling and they lose consciousness"*

*(R4-38 M*, *FGD 4)*

“*What I know [is] it is an infectious disease a person gets when he/she is young*

*(R3-55 M*, *FGD 4)*

*In addition*, *participants explained eclampsia as a disease that attacks from conception to the time of delivery*.

*"A disease that women get when they conceive to when they go for delivery*, *it can start slowly and worsen during the childbirth*, *that is how I know it"*

*(R3-32 M*, *FGD 2)*

Furthermore, some participants, both males and females, stated they were unaware of what eclampsia is. This increases the suspicion that some have never come across eclampsia or have never heard of it.

*“I don’t know*

*(R3-38 F*, *FGD5 & R8-38 F*, *FGD 5)”*

"*I don’t understand what eclampsia is all about*"

*(R1-42 M*, *FGD 6 & R4-32 M*, *FGD 6)*

"*I am not aware of anything; I normally hear about eclampsia but I am not aware of it*”

*(R4-25 F*, *FGD 5 & R5-28 F*, *FGD 5)"*

***ii) Signs and symptoms of eclampsia*.** Most participants were aware of the existence of eclampsia and had some knowledge related to its signs and symptoms. The majority of participants mentioned correct signs and symptoms including generalized edema, high blood pressure, severe headache, blurred vision and the body being weak.

*"What I think is proteinuria*, *swelling of legs*, *high blood pressure"*

*(R3-38 F*, *FGD 5)*

*"As far as I can see if the body swells*, *then the pressure becomes very high*, *then body weakness*, *because that leads to the eclampsia"*

*(R3-25 F*, *FGD 1)*

Nearly two-thirds of the study participants understand the signs and symptoms of eclampsia/preeclampsia, while the remaining one-third showed limited knowledge of signs and symptoms concerning eclampsia and mentioned that itching and weight loss, were among the signs and symptoms of eclampsia.

*"For sure I don’t…*, *you know no one has come to educate me on this*, *but the signs as we have said earlier is itching*, *she can tell you like I feel itching*, *in short*, *it doesn’t have a specific area”*

*(R7-50 M*, *FGD2 & R9-60 M*, *FGD 2)*

*"Weight loss*, *all that"*

*(R3-38 F*, *FGD 5)*

"*It’s dehydration*"

*(R2-49 F*, *FGD 5)*

In the same way, several male participants declared having no idea of the signs and symptoms of eclampsia.

*"No*, *I have never heard"*

*(R6-35 M*, *FGD 6)*

*"Even me here*, *I heard there is eclampsia but I don’t know its signs"*

*(R10-45 M*, *FGD 6)*

*“Yes*, *it’s like others said that mdudu*, *its signs are hard for sure*. *In short*, *we have a lack of knowledge about eclampsia"*

*(R1-45 M*, *FGD 4 & R9-30 M*, *FGD 6)*

***iii) Time of onset of eclampsia*.** More than half of the participants mentioned the correct gestational age at which eclampsia can occur.

*"You know*, *when you mention eclampsia*, *you have involved a period when a woman is pregnant*, *mostly it occurs when she is six*, *seven or eight months pregnant"*

*(R1-48 F*, *FGD 5*, *R3-55 M*, *FGD 4 & R1-24 F*, *FGD 1)*

"*I can say those are diseases that face women during delivery or after delivery*"

*(R7-50 M*, *FGD 2)*

Some of the participants responded incorrectly to the question of how eclampsia occurs. Most of these participants stated that eclampsia occurred at the moment of conception, that it can attack both men and women and that it can happen any time before, during or after pregnancy.

*"Yes*, *it affects women during conception*, *to some*, *it happened during early pregnancy and not on the third or fourth month of pregnancy"*

*(R3-55 M*, *FGD 4*, *R3-34 F*, *FGD 3 & R9-60 M*, *FGD 2)*

*"At any time not during pregnancy only*. *Some happen during pregnancy*, *while others before pregnancy*”

*(R6-29 F*, *FGD 5 & R1-24 F*, *FGD 1)*

*” Yes*, *during the evening that is when eclampsia happens”*

*(R1-45 M*, *FGD 4)*

However, the majority of male participants were not aware of many aspects of eclampsia. They mentioned eclampsia attacking anyone, male or female, and that it comes mostly during evening hours. Some were just informed of the condition; they did not know any details about it.

*“Yes*, *it can attack anyone*, *whether a woman or a man*, *to women it comes more during pregnancy and its time is mostly from six in the evening"*

*(R3-55 M*, *FGD 4)*

"*I don’t think I understand*"

*(R9-30 M*, *FGD 6)*

***iv) The alternative name of eclampsia*.** One of the research questions was to find out if there are alternative names for eclampsia used by the community members. Most of the participants, males and females, had the same common names for eclampsia (Mjusi/Mdudu). Mjusi and mdudu has the same meaning as eclampsia in community, depending on place of living, some respondents they called it mjusi and other they called it mdudu while they mean the same thing.

*"Yes*, *it’s there*, *mjusi (eclampsia)*. *We say mjusi because it’s like a devil that it can enter into the pregnant woman’s body without her knowing*, *it can affect also during the postpartum period"*

*(R9-60 M*, *FGD 2 & R9-47 F*, *FGD 3)*

*" Sometimes it happens*, *that is when we hear that she had eclampsia*, *but here in the village*, *we say mjusi*, *which means she got mjusi*, *there is another name for eclampsia that people use either mjusi or mdudu"*

*(R6-29 F*, *FGD 5 & R7-25 F*, *FGD 6)*

"*The whole area uses mdudu*"

*(R6-49 F*, *FGD 3*, *R1-48 F*, *FGD 5 & All*, *FGD 1)*

*"Maybe I should just say*, *I can say mdudu; in the community it mdudu but technically it eclampsia"*

*(R1-45 M*, *FGD 4)*

*"Mjusi*, *I have seen mjusi even our neighbour had mjusi"*

*(R1-24 F*, *FGD 1)*

***v) Perceived causes of eclampsia*.** Eclampsia was not perceived to be a condition specific to pregnancy. The community members had little understanding of eclampsia as a pregnancy complication. Nearly two-thirds of respondents did not believe that eclampsia was linked with high blood pressure while a few of them were not sure or did not believe that it was related to high blood pressure during pregnancy. However, all participants had the same explanations on perceived causes, which ranged from medical, and traditional to spiritual.

- **Perceived medical causes**

More than half of the female participants had a common understanding of the causes of eclampsia ranging from high blood pressure, family history of eclampsia, psychosocial stress and malaria.

“*Stress leads to blood pressure*, *which can cause eclampsia”*

*(R5-30 F*, *FGD1*, *R3-38 F*, *FGD 5 & R9-47 F*, *FGD 3)*

“*When a person inherits from parents then she can get eclampsia*”

*(R6-29 F*, *FGD 5)*

*“I think it’s a family stress*, *like from partners*, *in-laws or even the communities itself contributes so much*, *even stress due to the low economic status can cause eclampsia"*

*(R1-30 M*, *FGD6*, *R2-29 F*, *FGD 1& R7-38 M*, *FGD6)*

*"Yes*, *also malaria contributes to eclampsia"*

*(R8-46 F*, *FGD 3)*

Likewise, the majority of male participants confessed to being not exactly sure about the causes of eclampsia. It therefore, showed ambiguity, as it whether being anaemic, lacking vitamins and protein, dehydrated or malnutrition can cause eclampsia.

*"Maybe a mother has dehydration; that can lead to eclampsia"*

*(R10-45 M*, *FGD 6)*

*"We don’t know the causes of eclampsia; we think it could be due to anaemia*, *lack of vitamins and proteins"*

*(R2-42 M*, *FGD 6*, *R4-28 M*, *FGD 2 & R2-26 M*, *FGD 2*, *R5-42 M*, *FGD 2)*

- **Perceived traditional causes**

The majority of respondents perceived that eclampsia is related to mdudu/mjusi, (Satan or devil) and characterized by fits, tongue biting and being unconscious. Superstition, psychosocial stress, consumption of foods like tomato, octopus, and big fish, and eating slaughtered meat like chicken, cow and goat meat during pregnancy, are suspected to cause eclampsia in women. Nice-smelling lotion, soap and perfumes have been stated to attract the devil/Satan, especially pregnant women.

*"If a pregnant woman will eat octopus or tomatoes*, *these affect a pregnant woman"*

*(R8 M*, *40 FGD 2)*.

*"Yes*, *this fever has signs like the satanic*, *kind of*, *that is mdudu because your body becomes weak and you can’t do anything while you remain silent*…, *unable to talk"*

*(R7-71 M*, *FGD 4)*

*"Although soaps that smell*, *perfumes*, *are not good for a pregnant woman to use during bath"*

*(R5-30 F*, *FGD 1)*

*"And that’s why it was said that eating the slaughter meat during pregnancy*, *either it’s chicken or cattle*, *is prohibited*, *If you use it*, *you will get eclampsia"*

*(R1-24F*, *FGD 1)*

*"As said by other respondents*, *first food habits*, *like fish are the reasons for getting eclampsia "*

*(R7-50 M*, *FGD 2)*

*"At times it’s happed as superstitious"[belief]*

*(R8-46 F*, *FGD 3)*

*"But for sure*, *we know eclampsia*, *it’s more in superstitious things that are involved here"*

*(R3-55 M*, *FGD 4)*

- **Perceived spiritual causes**

Most of the male and female participants perceived women suffering from eclampsia as God’s wish and as something normal to happen to people. It can lead to death or survival and all that is God’s plan.

*"First*, *we see it as God’s wish*, *and then we see it as the normal thing to happen to a pregnant woman"*

*(R2-49 F*, *FGD 5 & R8-46 F*, *FGD 3)*

*"It’s God’s wish*, *which is how it is"*

*(R9-60 M*, *FGD 2)*

"*We know what happens spiritually*"

*(R9-60 M*, *FGD 2)*

***vi) Effects/complications*.** Most of the participants mentioned that eclampsia could lead to paralysis, premature delivery, mental or physical disability of the mother and even death of the mother and/or the baby.

*"There she can deliver a pre-mature baby due to [her] unhealthy condition that could contribute to eclampsia at seven months*, *eight months*, *nine months pregnancy"*

*(R1-48 F*, *FGD 5)*

*"As we said*, *death of a mother and child…but a child can be delivered pre-maturely"*

*(R1-48 F*, *FGD 5)*

*"She becomes paralyzed*, *either leg or arm or both"*

*(R9-52 F*, *FGD 3)*

"*The child can be born with brain dysfunction*"

*(R4-38 M*, *FGD 4)*

*Some male participants were not aware of the effects of eclampsia*.

*"Effects*, *I don’t know them*, *so eclampsia it may have happened but you never know its effects"*

*(R2-42 M*, *FGD 6)*

#### Theme 2: Perceived management and prevention of eclampsia

*i) Perceived management of eclampsia*. The study further wanted to investigate community members’ current management related to eclampsia. Most of the participants had different views on how a woman with eclampsia should be managed, including at the hospital level, traditional or spiritual practices.

- **Medical management towards eclampsia**

One-third of male and female participants mentioned medical practices that, they insisted on, like rushing patients to the hospital as the first step before doing anything to the mother.

Other participants mentioned taking traditional treatment.

*"The first thing is taking her to the hospital because we don’t know if it is eclampsia"*

*(R4-32 M*, *FGD 6)*

*"The first step is the hospital*, *after the hospital that is when I can do these other services*, *but at the beginning*, *there are those sheikhs*, *you can think of doctors who are close in the area for*, *like*, *advice*, *then…straight to the hospital for other services"*

*(R9-60 M*, *FGD 2)*

*"At the hospital where there are more experts*, *…because when she gets there*, *she had an intravenous infusion/drip but we have given first aid”*

*(R5-57 M*, *FGD 4)*

- **Traditional management towards eclampsia**

Home-based and traditional treatments for pregnancy complications were very common in all three regions. More than half of the participants mentioned several traditional remedies which are dried leaves used for eclampsia including, ("mfukuza jini"/monster chaser, "mnukamavi"/faeces smell leaves, majani ya mdimu /"lemon leaves", "kivumbasi”/lime basil", "mvunje"/leak leaves and "mbaazi"/dried peas leaves. Most of the participants believed that using smoke, fumes, drinking or steaming these traditional medicines would scare and chase away the "mdudu/mjusi (devil)" as a way of treating or preventing eclampsia.

*"Medicine*, *you can find like dried peas leaves"*

*(R7-33 F*, *FGD 5)*

*"We use our traditional medicine*, *like applying oil*, *garlic*. *Garlic*, *traditionally*, *is to calm her then after*, *another treatment follows "*

*(R9-30 M*, *FGD 6)*

"*She should be using bad smell oil so that mdudu runs away*"

*(R3-34 F*, *FGD 3)*

*"You grind garlic and leak leaves*, *you give her some to drink and smear the other one to chase away mjusi"*

*(R2-29 F*, *FGD 1)*

*"Because it happened to me*, *luckily my mother was there she used fumes of monster chaser on me and I got relief*, *not bad I thank God"*

*(R8-28 F*, *FGD 5)*

*"We even use animal skin*, *we can also use a snake or lizard skin for treating"*

*(R9-60 M*, *FGD 2)*

*"You must start with what will help*, *I am telling you that there are people who started with those traditional medicines till the end of childbirth*, *there is a tree that is very good I will tell you its name it’s called mnukamavi"*

*(R6-59 M*, *FGD 2)*

*"The first thing is we find lemon leaves*, *we mix with lime basil and give her to drink till we find a car we take her to the hospital"*

*(R6-49 F*, *FGD 3)*

"*We also use traditional fumes and we apply kerosene on her back*"

*(R7-48 F*, *FGD 3)*

- **Spiritual management towards eclampsia**

In terms of spiritual practices, all participants mentioned makombe (writing Quraan on a piece of paper that is immersed in water and given to the patient either for drinking and/or applying on the body) that can be used to treat eclampsia and involves reading a holy book like the Quraan.

*"Yes*, *I took water in a glass*, *I took my time to read Quran and she was calm*, *so that is when we said at times it spiritual and at times it scientific"*

*(R2-42 M*, *FGD 6)*

*"Makombe*, *it’s a written Quran [It’s a verse of holy Qu- ran written on a piece of paper and when it dries you soak it in a bowl of water and they usually drink or apply that water to the body]”*

*(R3-34 F*, *FGD 3)*

"*You are also given Quran writings for steaming*"

*(R4-35 F*, *FGD 1)*

*"There is alternative treatment because*, *at that time many Zanzibarians*, *we can say perhaps this is local*, *you will find makombe for a person and other herbs to continue with”*

*(R6-35 M*, *FGD 6)*

*ii) Perceived prevention of eclampsia*. Nearly one-third of participants explained that early attendance of the clinic by pregnant women, avoiding stress at the family level and men being close and supporting their wives during pregnancy help a mother to avoid developing eclampsia.

*"A pregnant mother should attend the antenatal clinic on time*, *it’s obvious that we should discard these traditional thoughts on eclampsia”*

*(R1-48 F*, *FGD 5)*

*"Also fear of being pregnant also contributed*, *so husbands should be polite and support their wives during pregnancy to avoid fear"*

*(R4-28 M*, *FGD 2)*

*"To follow hospital procedures*, *like attending an antenatal clinic for treatment*, *and mother should reduce salt intake"*

*(R3-55 M*, *FGD 4)*

However, the majority of all participants mentioned that they used traditional remedies and herbs to prevent eclampsia in their community. They also explained about avoiding perfumes for pregnant women, doing home activities like cooking and fetching water during pregnancy, and using herbal drugs and mafusho. They insisted on having lemons at all times during pregnancy to prevent eclampsia.

*"Pregnant women should avoid using perfumes and use mafusho only”*

*(R2-43 F*, *FGD 3)*

*"She should not do any hard work*, *there must be a person to help her like for cooking*, *fetching water*, *if the food is there*, *then should be brought to her"*

*(R8-40 M*, *FGD 2)*

*"That is why they are protected*, *like lemon has to be there*, *avoid perfumes because those will stir up mdudu to come to you"*

*(R3-55 M*, *FGD 4)*

*"You don’t have to use perfume when you are pregnant*, *when a woman knows herself as being pregnant you should not use perfumes/nice smelling lotion or oil"*

*(R1-24 F*, *FGD 1 & R2-43 F*, *FGD 3)*

### Participants’ recommendations

Finally, participants gave their recommendations to prevent eclampsia and save the lives of pregnant women and their children. Investing in educating communities was the most important recommendation. Setting a special time and venue for education will help largely in the prevention of eclampsia in the community setting.

*"I repeat we don’t know eclampsia"*

*(R1-48 F*, *FGD 5)*

*"First this discussion should have a special time at a special place if it can be planned*, *should also reach many people*, *as most of them don’t understand eclampsia"*

*(R1-33*, *M FGD 2)*

## Discussion

The study aimed to explore knowledge and perception towards eclampsia among women aged 18–49 years and men in Unguja. This is the first qualitative study broadly exploring knowledge of eclampsia, its local names, perceived causes and consequences as well as perceived management and prevention of eclampsia by communities in Unguja, Zanzibar.

Our study found that there was a lack of understanding of eclampsia in all three regions of Unguja Island. The majority of participants did not realize/know that eclampsia was related to hypertensive disorder in pregnancy, and those who did recognize the relationship often did not know its signs and symptoms, its causes and effects and when it occurs. Most participants believe in traditional and spiritual treatment. Communities reported several traditional remedies used for the treatment of eclampsia.

The results of this study are similar to the studies that have been conducted in the Mtwara and Dodoma regions and the Same district in Tanzania found that more than half of the respondents were not aware of eclampsia and its complications [1, 14, 16]. These similarities might be due to similarities in cultural and geographical features of the locations where the studies were conducted.

Despite the unawareness of eclampsia observed in the current study, participants managed to correctly mention the complications arising from eclampsia. Concerning the effects of eclampsia, these findings are similar to the studies done in Ethiopia; most participants were aware of the seriousness of preeclampsia/eclampsia for the health of both mother and fetus [11]. In addition, our results are similar to studies done in Mozambique, India and Nigeria, where most participants mentioned that death of the mother and/or infant, preterm delivery and operative delivery were the common complications mentioned by the participants” [12, 20, 21].

Our study revealed only partial awareness and knowledge of eclampsia. This finding supports the need to reinforce education programs regarding signs and symptoms, causes, occurrence and treatment of this condition. Although some participants were aware of the consequences of eclampsia, they did not always view eclampsia as associated with pregnancy. This was illustrated by the fact that many different local names for the disease were mentioned, which do not relate to pregnancy or pregnant women. Eclampsia is generally viewed as caused by supernatural forces, such as Mdudu or Mjusi, meaning a disease of the "devil”. The present findings seem to be in line with the study done in the Mtwara region of Tanzania which found that, nearly half of the participants mentioned that, eclampsia is caused by the devil spirit [1]. In a study in Mozambique, [22] the local name for eclampsia was “mavabji ya nweti” (illness of the moon). The results of this study differ from previous studies done in Pakistan, India and Nigeria where eclampsia was known as epilepsy of pregnancy, seizures, paralysis or infection [10, 13, 21]. Evil spirits, which were mentioned by participants in our study, were not mentioned in these studies.

In our study, social and emotional problems were perceived as the leading causes of eclampsia. This is comparable to what was previously found in studies done in Asia and Africa. In India and Pakistan, participants mentioned that excessive thinking or stress, particularly related to marital problems, lack of rest, domestic problems, the burdens of household chores, diet, tension and social responsibilities were the most common causes of high blood pressure in pregnancy, leading to eclampsia [9, 16]. In a qualitative study done in Nigeria and Mozambique, [12, 19], women also suggested that marital problems, such as mistreatment by in-laws, excessive thinking or worry, anger and sadness, heredity, diet, and depressive thoughts/stress, cause eclampsia. In a study from Ethiopia, however, respondents mentioned lack of physical activity, being overweight, being primigravidae and failure to have regular ANC follow-up as some of the attributed risk factors of preeclampsia/eclampsia [11].

This study also revealed several misperceptions rooted in traditional and spiritual beliefs that ultimately shape the way people act and react, including not touching women or assisting women when they get eclamptic fits to avoid becoming affected. These misconceptions, as well as the use of traditional treatments, can lead to serious delays in seeking care at the health facility, decreasing the chances of survival when help is eventually sought at the health facility. People tend to first seek traditional remedies such as holy water, herbs, drinks and steaming to alleviate eclampsia.

The findings from this study illustrated the misconceptions surrounding eclampsia among community members. The insights provided can help the identification of converging and contrasting views between the local and the biomedical perspectives. This improved understanding can then inform the development of more appropriate health promotion messages for pregnant women, their family members and communities. This information can be used to organize health education programs for the communities, to improve their awareness of sociocultural factors contributing to maternal morbidity and mortality in the regions they serve.

### Strengths and limitations of the study

This is the first study in Unguja Island, Zanzibar, to describe the perception of eclampsia in the community setting. The data collectors, including the PI, were very close to the communities, which made the discussion more interactive; participants felt free to contribute. The majority (88%) of study participants had completed secondary education which is higher compared to national data of lower secondary completion among Tanzania’s which ranges between 32% in men and 35%i in women, this might mean they had greater knowledge of eclampsia than people with lesser education, this finding could be due to the limited sample size and the selection biases, which might cause the potential bias. Furthermore, using this study’s findings will help to continue to the next stage of research that will contribute to improving maternal and child health.

### Conclusion and recommendations

It is obvious that eclampsia is not well-known to the community in Zanzibar; however, hypertension and seizures are perceived as conditions that may occur before and during pregnancy or after childbirth. The majority of the participants had a lack of understanding regarding eclampsia and perceived it as a traditional issue, herbs and traditional remedies were used to prevent eclampsia in their community. These findings highlight the necessity for additional education within the community regarding eclampsia and its contributing factors. Therefore, there is a need for improved community education programs and awareness campaigns about eclampsia during antenatal care (ANC) visits. These efforts are essential to supplement and correct any misperceptions regarding the causes and treatment of eclampsia. This is crucial for preventing complications associated with eclampsia and ensuring the effective implementation of strategies aimed at reducing morbidity and mortality among mothers and babies in Zanzibar. Furthermore, more representative studies, which will help to define appropriate strategies and participative operational research are warranted to gather additional information on eclampsia in both community and health facility settings.

## Supporting information

S1 File (DOCX)
