# Supplementary material for: Knowledge of and perception towards eclampsia among women and men in Unguja Island, Zanzibar: A qualitative study
Source: PLoS One. 2025 Jan 15;20(1):e0313536. doi: 10.1371/journal.pone.0313536 (PMC11734952; doi:10.1371/journal.pone.0313536)
Supplement: S1 File — (DOCX) [file pone.0313536.s001.docx]

**The Interview Guide** **- Knowledge of and Perception Towards Eclampsia**

**Introduction**

- Introduce the research team
- Introduce the study topic
- Take the written consent of participation and willingness to participate
- Explain the aims and objectives of the study
- Explain confidentiality and anonymity
- Explain the recording (audio-recorded), the length of (50-60 minutes), and the nature of the discussion.
- Check if participants have any questions before starting the discussion

**General rules:**

- Questions should be broad and open-ended
- Don’t assume to understand what participants describe. Use clarifying questions

**Knowledge and Perceived Causes towards Eclampsia Questions**

1. Can you please tell me what you understand about the word eclampsia? *Please explain*

*Probe more (i.e., including those that occur during pregnancy, delivery and post-delivery)*

1. What are the common signs and symptoms that the woman shows when she has eclampsia? *Please explain more, (Probe more questions on danger signs).*
2. How does eclampsia occur? *(Probe more questions, i.e., including those that occur during pregnancy, delivery and post-delivery)*
3. Are there any other words used for eclampsia? *Please can you mention them*
4. Could you tell me more about the causes of eclampsia? (*Probe for a complete list of causes and understanding of risk factors).*
5. What effects do you think, could the mother and the baby might get due to eclampsia? *Probe more on maternal and neonatal outcomes, during pregnancy, intrapartum and the postnatal period.*

**Perceived management and prevention of eclampsia questions**

7. What do you do when you first think the woman has eclampsia?

8. (Do we treat eclampsia in our community, and if so, how do we treat it?), *Probe more (on the medicine used, place of used to treat the woman e.g., at home, to the traditional healer, traditional birth attendants, to hospital, in each probe also probes why and why not)*

1. Is there anything you can do to avoid getting eclampsia? *Please tell me how you can prevent it, Probe more on management and preventive measures.*

**summary**

- Before summarising some of the key points from our discussion. Is there anyone with anything else who wants to share?
- Are there any other points that you would like to discuss?
- Do you have any questions concerning what we have discussed?
- Thank You for Your Cooperation.

**The COREQ (Consolidated criteria for Reporting Qualitative research) Checklist**

| **Topic** | **Item No.** | **Guide Questions/Description** | **Reported on Page No.** |
| --- | --- | --- | --- |
| **Domain 1: Research team and reflexivity** | | | |
| *Personal Characteristics* |  |  |  |
| Interviewer/facilitator | 1 | Which author/s conducted the interview or focus group? | 5 |
| Credentials | 2 | What were the researcher’s credentials? E.g. PhD, MD | Title page |
| Occupation | 3 | What was their occupation at the time of the study? | Title page |
| Gender | 4 | Was the researcher male or female? | Not stated |
| Experience and training | 5 | What experience or training did the researcher have? | Title page |
| *Relationship with participants* | | | |
| Relationship established | 6 | Was a relationship established prior to study commencement? | 6 |
| Participant knowledge of the interviewer | 7 | What did the participants know about the researcher? e.g. personal goals, reasons for doing the research | 5 - 6 |
| Interviewer characteristics | 8 | What characteristics were reported about the interviewer/facilitator? e.g. Bias, assumptions, reasons and interests in the research topic | 21 |
| **Domain 2: Study design** | | | |
| *Theoretical framework* |  |  |  |
| Methodological Orientation and Theory | 9 | What methodological orientation was stated to underpin the study? e.g.  grounded theory, discourse analysis, ethnography, phenomenology, content analysis | 5 |
| *Participant selection* |  |  |  |
| Sampling | 10 | How were participants selected? e.g. purposive, convenience, consecutive, snowball | 5 |
| Method of approach | 11 | How were participants approached? e.g. face-to-face, telephone, mail, email | 6 |
| Sample size | 12 | How many participants were in the study? | 5 |
| Non-participation | 13 | How many people refused to participate or dropped out? Reasons? | 8 |
| *Setting* |  |  |  |
| Setting of data collection | 14 | Where was the data collected? e.g. home, clinic, workplace | 5 |
| Presence of nonparticipants | 15 | Was anyone else present besides the participants and researchers? | 5 |
| Description of sample | 16 | What are the important characteristics of the sample? e.g. demographic data, date | 8 |
| *Data collection* |  |  |  |
| Interview guide | 17 | Were questions, prompts, and guides provided by the authors? Was it pilot-tested? | 6 |
| Repeat interviews | 18 | Were repeat interviews carried out? If yes, how many? | 0 |
| Audio/visual recording | 19 | Did the research use audio or visual recording to collect the data? | 6 |
| Field notes | 20 | Were field notes made during and/or after the interview or focus group? | 6 |
| Duration | 21 | What was the duration of the interviews or focus groups? | 6 |
| Data saturation | 22 | Was data saturation discussed? | 7 |
| Transcripts returned | 23 | Were transcripts returned to participants for comment and/or | 6 |
| **Topic** | **Item No.** | **Guide Questions/Description** | **Reported on Page No.** |
| **Domain 3: analysis and findings** | | |  |
| *Data analysis* |  |  |  |
| Number of data coders | 24 | How many data coders coded the data? | 7 |
| Description of the coding tree | 25 | Did the authors provide a description of the coding tree? | 9 |
| Derivation of themes | 26 | Were themes identified in advance or derived from the data? | 7 |
| Software | 27 | What software, if applicable, was used to manage the data? | 7 |
| Participant checking | 28 | Did participants provide feedback on the findings? | 18 |
| *Reporting* |  |  |  |
| Quotations presented | 29 | Were participant quotations presented to illustrate the themes/findings?  Was each quotation identified? e.g. participant number | 7 - 18 |
| Data and findings consistent | 30 | Was there consistency between the data presented and the findings? | 7 - 18 |
| Clarity of major themes | 31 | Were major themes clearly presented in the findings? | 7 - 18 |
| Clarity of minor themes | 32 | Is there a description of diverse cases or discussion of minor themes? | 7 - 18 |

Developed from: Tong A, Sainsbury P, Craig J. Consolidated criteria for reporting qualitative research (COREQ): a 32-item checklist for interviews and focus groups. *International Journal for Quality in Health Care*. 2007. Volume 19, Number 6: pp. 349 – 357
